# Population-based prevalence and screening gaps of hepatitis C, B and D, Germany, 2020 to 2021

**DOI:** 10.2807/1560-7917.ES.2026.31.25.2500864

**Published:** 2026-06-25

**Authors:** Zahra Ghodratian, Katharina Grikscheit, Loraine Busetto, Benjamin Marx, Niko Kohmer, Arne Auste, Raphael Scheu, Manuela Harries, Vanessa Melhorn, Thomas Illig, Monika Strengert, Annemarie Berger, Berit Lange, Sandra Ciesek

**Affiliations:** 1 Institute for Medical Virology, University Hospital Frankfurt, Goethe University Frankfurt, Frankfurt am Main, Germany; 2 Helmholtz Centre for Infection Research, Department of Epidemiology, Braunschweig, Germany; 3 Hannover Unified Biobank, Hannover Medical School, Hannover, Germany; 4 German Centre for Infection Research (DZIF), TI Bioresources, Biodata and Digital Health, Braunschweig, Germany; 5 German Centre for Infection Research (DZIF), Braunschweig, external partner site Frankfurt, Germany; 6 Fraunhofer Institute for Translational Medicine and Pharmacology (ITMP), Frankfurt am Main, Germany

**Keywords:** Hepatitis B virus, Hepatitis C virus, epidemiology, screening, viral hepatitis

## Abstract

**BACKGROUND:**

Chronic hepatitis C (HCV) and hepatitis B (HBV) viruses remain major causes of liver-related morbidity and mortality. Despite a low prevalence in Germany, migration from higher-endemic regions and screening gaps may hinder progress towards World Health Organization (WHO) 2030 elimination targets. Population-based data are needed for surveillance, screening and elimination.

**AIM:**

We aimed to estimate prevalence of HCV, HBV and hepatitis D virus (HDV) in adults in Germany.

**METHODS:**

We retrospectively analysed 10,000 serum samples from the Multilocal and Serial Prevalence Study of Antibodies against SARS-CoV-2 in Germany (MuSPAD, 2020–2021), by age and gender distribution. Samples were pooled and tested for HCV and HBV by PCR, for HBV surface antigen (HBsAg) and for antibodies against HCV and HBV. Samples positive for HBsAg were analysed for HDV.

**RESULTS:**

Active HCV infection (HCV RNA) was detected in four (0.04%; 95% CI: 0.02–0.1) persons. Seroprevalence for HCV was 0.17% (95% CI: 0.11–0.27), highest among 60–69-year-olds (0.53%). Active HBV infection (HBV DNA and/or HBsAg) was detected in 21 participants (0.21%; 95% confidence interval (CI): 0.14–0.32), peaking at 0.44% among 30–39-year-olds. No HDV infection was confirmed.

**CONCLUSION:**

Recognising that relevant high-risk groups may be underrepresented in our data, the overall HCV and HBV prevalence in Germany appears low but non-negligible. Adults under 35 years are not covered by the national health check programme for all adults aged ≥ 35 years, suggesting potential screening gaps. Systematic HDV testing among HBV-positive individuals and targeted testing strategies may support progress towards WHO elimination goals.

**Key public health message**

**What did you want to address in this study?**

Infection with hepatitis C (HCV), B (HBV) and D (HDV) virus can lead to a chronic liver disease. We aimed to update estimates of hepatitis C, B and D prevalence in Germany, as previous data were largely based on blood donors or selected groups and therefore may not represent the general population.

**What have we learnt from this study?**

Hepatitis C, B and D are not widespread in Germany, but they still affect some people. Among the 10,000 people we tested, we found four people with an active HCV infection and 13 people with a past HCV infection. We also found 21 persons with an HBV infection, none of whom were confirmed infected with HDV. Importantly, some people with hepatitis did not know they were infected, showing that not all cases are currently being diagnosed.

**What are the implications for public health practice?**

Hepatitis C, B and D infections are uncommon in Germany. However, some infections remain undiagnosed, so targeted testing is important. Expanding testing, ensuring that people diagnosed with hepatitis B are also tested for hepatitis D, and improving linkage to care can help close gaps and support the goal of eliminating viral hepatitis.

## Introduction

Viral hepatitis remains a major global health challenge, accounting for an estimated 1.3 million deaths in 2022 due to chronic hepatitis B virus (HBV) or hepatitis C virus (HCV) infections and their sequelae, such as cirrhosis and hepatocellular carcinoma [1]. Worldwide, around 304 million people live with chronic HBV or HCV infection and approximately 2 million new infections occur each year [1]. Effective tools to reduce this burden are available: direct-acting antivirals (DAAs) for HCV achieve cure rates above 95% [2,3], while prophylactic vaccination against HBV offers long-term protection [4]. These advances raise realistic prospects of reducing morbidity and mortality related to viral hepatitis, particularly in countries with adequate access to healthcare.

In 2022, the World Health Organization (WHO) adopted a global health sector strategy aiming to eliminate HBV and HCV as public health threats by 2030 [5]. However, many infections remain undiagnosed as they often progress asymptomatically until advanced liver disease develops [6,7]. Globally, only ca 13% of people living with HBV and 36% of people living with HCV are estimated to know of their infection [8,9]. In Europe, fewer than half of HCV-infected individuals are aware of their infection [10,11]. According to the European Centre for Disease Prevention and Control (ECDC), ca 65% of people living with HBV are estimated to remain undiagnosed, underscoring the relevance of underreporting due to underdiagnosis [12,13]. Surveillance and screening are therefore critical yet approaches and data quality vary substantially between countries [14-17].

Germany is considered a low-prevalence country for HBV and HCV [18,19]. Earlier population-based data from the German Health Interview and Examination Survey for Adults (DEGS1, 2008–2011) estimated HBV (hepatitis B virus surface antigen (HBsAg)) and anti-HCV seroprevalence, each, at 0.3% [20]. The DSGS survey by the German national public health institute repeatedly collected representative data on the health status of adults residing in 180 German cities and municipalities. More recent blood donor surveillance (2017–2019) reported even lower prevalence (HBV: 0.072–0.061%; HCV: 0.050–0.041%) [19]. However, due to regulations related to pre-donation screening, blood donors represent a younger and healthier subpopulation than the actual German population, likely underestimating true prevalence. In contrast, data from a 2021–2022 national screening programme in primary care (Check-up 35 +) indicated higher rates (HBsAg: 0.54%, HBV DNA: 0.39%, anti-HCV: 0.79%, HCV RNA: 0.13%) [21]. Uptake of the general health check-ups offered by the German health system (including the 35 + programme) is comparable to or slightly higher than in other countries, such as the United Kingdom (UK) or Austria. Uptake was also higher among individuals who already had frequent contact with the healthcare system, but lower among populations with higher health risks and lower healthcare utilisation [22]. These discrepancies highlight the need for updated population-based prevalence estimates beyond specific subgroups.

Recent developments may impact prevalence trends in opposite directions: the availability of DAAs and universal HBV vaccination should reduce chronic infections, while migration from higher-prevalence regions and possible disruptions to prevention programmes during the COVID-19 pandemic may have contributed to stable or even rising rates. While the pandemic has globally impacted preventive infectious disease programmes [23], evidence from high-income countries like Germany is lacking and mainly restricted to prevention programmes of non-communicable diseases like cancer [24]. Whether the overall prevalence of HBV and HCV in Germany is declining, stagnating or shifting within specific subgroups, therefore, remains unclear.

In addition, co-infection with hepatitis D virus (HDV) substantially increases the risk of severe liver disease, yet population-based data for Germany remain scarce. Understanding the prevalence of HDV among HBV-infected individuals is important for clinical management and elimination strategies.

Reliable estimates of HBV and HCV prevalence are essential for guiding screening, vaccination and elimination strategies. In Germany, previous prevalence estimates have largely been based on surrogate populations such as blood donors with limited generalisability. Against this background, we aimed to estimate the prevalence of HCV, HBV and HDV in Germany. To our knowledge, this study provides the first contemporary, population-based estimates of HBV and HCV prevalence in Germany since the widespread implementation of DAAs. These data are intended to inform surveillance efforts, guide screening strategies and support Germany’s progress towards the WHO 2030 elimination targets.

## Methods

### Study setting and patient population

The study involved retrospective and random selection of 10,000 pseudonymised serum samples from the MuSPAD (Multilocal and Serial Prevalence Study of Antibodies against SARS-CoV-2 in Germany) cohort; a large, population-based, cross-sectional seroepidemiological study designed to assess SARS-CoV-2 antibody prevalence in adults aged over 18 years in the German population early in the COVID-19 pandemic [25]. In seven regions across Germany, from July 2020 to August 2021, participants were randomly selected from population registers and invited by postal mail to participate in the MuSPAD cohort. Those who provided written consent and did not have contraindications for giving a blood sample were eligible for the study [25]. Participants were invited for blood sampling and completed a questionnaire on demographic, socioeconomic and health-related factors [25]. The detailed study design has been described previously [25].

In 2022, the ethical approval for the MuSPAD cohort was adapted to allow for retrospective testing of already collected serum samples against other infections as well [26]. This included pathogens causing not only acute, but also chronic infectious diseases, such as HBV and HCV, to provide updated population-based prevalence estimations that are not limited to specific cohorts, such as blood donors or high-risk populations. The ethical approvals covered the possibility to contact MuSPAD participants when diagnosed, for example, with a chronic hepatitis infection. These phone calls were performed by medical doctors of the MuSPAD study team who provided information about the positive test and offered referrals to appropriate medical services. They also asked if the participant had previously been aware of their diagnosis and whether they were currently receiving treatment.

We followed key elements of the STROBE reporting frameworks for population-based observational research to ensure transparent and standardised reporting of epidemiological findings [27].

### Pooling strategy

We defined active HBV infection as the presence of HBsAg with or without detectable HBV DNA. Active HCV infection was defined as detectable HCV RNA. Past HCV exposure was defined as detection of antibodies against HCV in the absence of detectable HCV RNA. The study samples were organised within Hannover Unified Biobank (HUB) at the Hannover Medical School (MHH) and stored in boxes containing 96 uniquely barcoded tubes. For this study, 48 samples per box were pooled (50 µL per sample; total volume 2,400 μL; 1:48 dilution) and subsequently analysed. Given the analytical sensitivity of the assays, clinically relevant viral loads are expected to remain detectable despite dilution. Pools tested positive in PCR or antigen testing were further resolved by retesting in smaller sub-pools of six samples each, followed by individual testing of samples from positive sub-pools. All positive samples were additionally retested for confirmation. Detailed information on pooling strategies, spiking procedures and monitoring of anti-HBs-IgG are available in Supplementary Methods.

### Detection of hepatitis B and C virus with quantitative PCR

All pools, as well as individually analysed samples, were tested for HBV DNA and HCV RNA using the automated Alinity m System with the corresponding HBV and HCV assays (Abbott Molecular, Des Plaines, the United States (US)) following the manufacturer’s instructions. Information about limits of detection and genotyping, including primer sequences, are presented in Supplementary Methods.

### Serological testing for hepatitis B, C and D virus

Serological analyses for antibodies against HBV and HCV were performed using the Abbott Alinity i (Abbott Molecular) according to the manufacturer’s specifications [28]. For IgG and IgM antibodies against HCV, samples over cut-off 1 were considered positive. For detection of HBsAg, cut-off was > 1. For IgG antibodies against HBsAg, samples with  > 10 mIU/mL were considered positive. For IgG antibodies against core antigen (HBc), samples > 1 were considered positive.

The samples with a negative HBsAg (below detection level) were not tested against HDV antibodies or RNA. Samples positive for HBsAg (referred to as “HBV positive”, i.e. patients #1–14) were individually examined for the presence of antibodies to HDV. If enough sample material was available, we tested HBsAg-positive samples for HDV antibodies. Testing for IgG antibodies against HDV was done using the fully automated chemiluminescent immunoassay (CLIA) LIAISON XL MUREX Anti-HDV (DiaSorin, Dietzenbach, Germany) according to the manufacturer’s instructions.

### Detection of hepatitis D virus with quantitative PCR

To rule out HDV co-infection, we tested HBsAg-positive samples for HDV. The samples were tested individually, but each sample was diluted with negative plasma in different ratios (four times 1:4 and once 1:10). Viral RNA was extracted from 400 µL of serum using the INSTANT Virus RNA/DNA Kit (Roboscreen, Leipzig, Germany). Detection and quantification were carried out using the RoboGene HDV RNA quantification kit (Roboscreen), version 2.0 on the Applied Biosystems 7500 Fast Real-Time PCR System (Thermofisher, Waltham, US), according to the manufacturer's instructions. This assay has a LOD of 6 IU/mL with a linear range of 5 to 10^8^ IU/mL [29]. Positive samples were sent for genotyping as confirmation and to exclude any contamination. More information including primer sequences are available in Supplementary Methods.

### Statistical analysis

Prevalence estimates and their confidence intervals (CI) were calculated at the 95% level using the Wilson score method [30]. Prevalence of HCV/HBV infection was determined as the number of RNA/DNA-positive participants (as tested above the limit of detection (LOD)) per 10,000 samples and HCV/HBV seroprevalence was defined as the number of anti-HCV or HBsAg positive per 10,000. Subgroup analyses were conducted descriptively, including stratification by age groups and gender. Differences between groups were explored using chi-square or Fisher’s exact test where appropriate. Statistical significance was set at p < 0.05 (two-sided). Statistical analyses were performed with R version 4.6.0 (https://www.r-project.org/). Figures and graphical representations were generated with Canva and GraphPad Prism (version 10.2.3, GraphPad Software; https://www.graphpad.com/).

## Results

### Prevalence of hepatitis C virus

**Figure 1:**
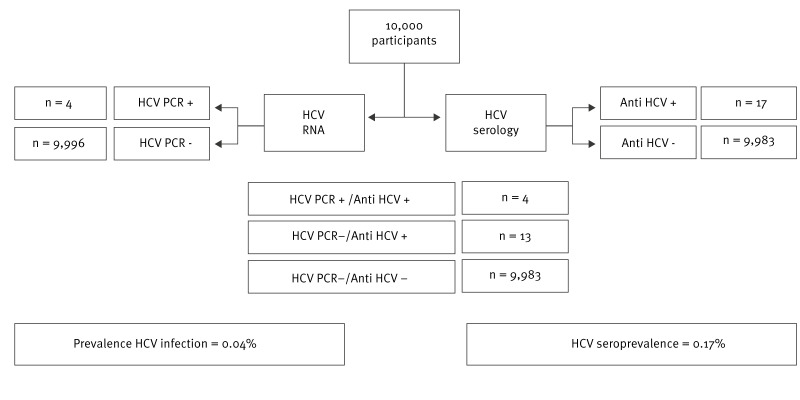
Workflow for testing of serum samples for hepatitis C virus from participants of the MuSPAD cohort, Germany, 2020–2021 (n = 10,000)

Figure 1 illustrates the testing workflow and resolution of positive pools. After resolution of positive pools and confirmatory individual testing, we detected HCV-RNA in four individuals determining a prevalence in the cohort of 0.04% (95% CI: 0.02–0.1) (Figure 1, Table 1, Table 2).

HCV: hepatitis C virus; MuSPAD cohort: Multilocal and Serial Prevalence Study of Antibodies against SARS-CoV-2 in Germany.

**Table 1:** Demographic and virological characteristics of participants of the MuSPAD cohort infected with hepatitis C virus, Germany, 2020–2021 (n = 17)

ID	Age (years)	Gender	HCV-PCR (IU/mL)	Genotype	Anti-HCV (S/CO)
1	60–69	Man	1,089,200	1a	20.97
2	30–39	Man	830,880	3a	17.85
3	≥ 80	Woman	2,252,740	1b	19.68
4	40–49	Woman	98,680	NA^a^	17.76
5	60–69	Woman	Negative	NA	17.48
6	60–69	Man	Negative	NA	15.97
7	60–69	Man	Negative	NA	15.19
8	50–59	Woman	Negative	NA	18.23
9	60–69	Woman	Negative	NA	3.43
10	40–49	Man	Negative	NA	17.25
11	60–69	Woman	Negative	NA	14.50
12	30–39	Woman	Negative	NA	17.33
13	60–69	Woman	Negative	NA	18.34
14	40–49	Woman	Negative	NA	16.09
15	60–69	Woman	Negative	NA	15.56
16	60–69	Woman	Negative	NA	14.36
17	60–69	Man	Negative	NA	18.60

HCV: hepatitis C virus; ID: identification code; MuSPAD cohort: Multilocal and Serial Prevalence Study of Antibodies against SARS-CoV-2 in Germany; NA: not applicable; S/CO: signal/cut-off.

^a^ Amount of sample material insufficient for genotyping.

**Table 2:** Prevalence of hepatitis B and C viruses among participants of the MuSPAD cohort, by age and infection status, Germany, 2020–2021 (n = 10,000)

Age (years)	n	Man		Woman		Diverse^a^		NA		Anti-HCV			HCV-RNA			HBV infection^b^		
		n	%	N	%	n	%	n	%	n	%	95% CI	n	%	95% CI	n	%	95% CI
Total	10,000	4,386	43.86	5,586	55.86	2	0.02	26	0.26	17	0.17	0.11–0.27	4^c^	0.04	0.02–0.1	21	0.21	0.14–0.32
18–19	115	46	40.00	69	60.00	0		0		0	0	0–3.23	0	0	0–3.23	0	0	0–3.23
20–29	1,188	483	40.66	704	59.26	1	0.08	0		0	0	0–0.32	0	0	0–0.32	1	0.08	0.01–0.48
30–39	1,576	661	41.94	913	57.93	1	0.06	1	0.06	2	0.13	0.03–0.46	1	0.06	0.01-0.36	7	0.44	0.22–0.91
40–49	1,413	618	43.74	795	56.26	0		0		3	0.21	0.07–0.62	1	0.07	0.01–0.4	2	0.14	0.04–0.51
50–59	2,198	944	42.95	1,254	57.05	0		0		1	0.05	0.01–0.26	0	0	0–0.17	6	0.27	0.13–0.59
60–69	1,891	838	44.32	1,053	55.68	0		0		10	0.53	0.29–0.97	1	0.05	0.01–0.3	2	0.11	0.03–0.38
70–79	1,089	552	50.69	537	49.31	0		0		0	0	0–0.35	0	0	0–0.35	3	0.28	0.09–0.81
> 80	452	220	48.67	232	51.33	0		0		1	0.22	0.04–1.24	1	0.22	0.04–1.24	0	0	0–0.84
NA	78	24	30.77	29	37.18	0		25	32.05	0	0	0–4.69	0	0	0–4.69	0	0	0–4.69

HBV: hepatitis B virus; HCV: hepatitis C virus; MuSPAD cohort: Multilocal and Serial Prevalence Study of Antibodies against SARS-CoV-2 in Germany; NA: information about gender not available.

^a^ In Germany, diverse is a legally recognised category for people who are not exclusively male or female, translating approximately to non-binary, another gender, gender-diverse or intersex. It is used in official forms and identification documents alongside male and female and offers a third option.

^b^ Participants were considered infected with HBV if HBV DNA and/or hepatitis B surface antigen (HBsAg) were detected.

^c^ These patients had also antibodies against HCV.

**Figure 2:**
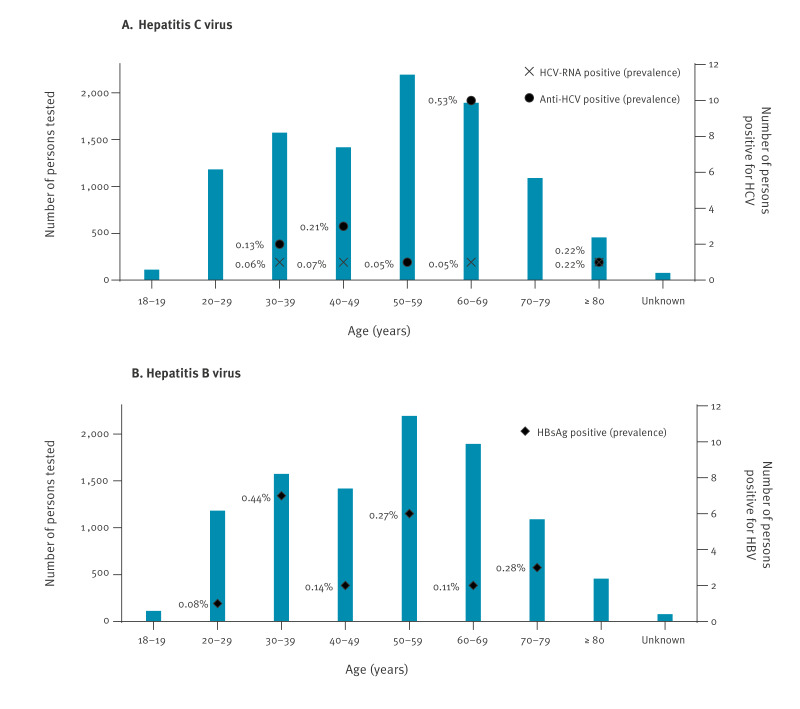
Age distribution of participants of the MuSPAD cohort tested for hepatitis C and B virus, Germany, 2020–2021 (n = 10,000)^a^

HBsAg: Hepatitis B surface antigen; HCV: hepatitis C virus; MuSPAD cohort: Multilocal and Serial Prevalence Study of Antibodies against SARS-CoV-2 in Germany.

^a^ Largest age groups: 50–59 years (n = 2,198) and 60–69 years (n = 1,891).

Antibodies against HCV were identified in samples from 17 participants, and HCV RNA was detected in four of these. Thus, the overall seroprevalence was 0.17% (95% CI: 0.11–0.27) (Figure 1, Table 1, Table 2). The median age of the participants infected with HCV was 63 years (range: 36–80 years). Eleven of these 17 persons were women. In the total cohort, median age was 53 years (range: 18–99 years) and 55.9% were women. The highest HCV seroprevalence was measured among 60–69-year-olds (0.53%; 95% CI: 0.29–0.97) (Table 2, Figure 2A).

### Prevalence of hepatitis B virus

**Figure 3:**
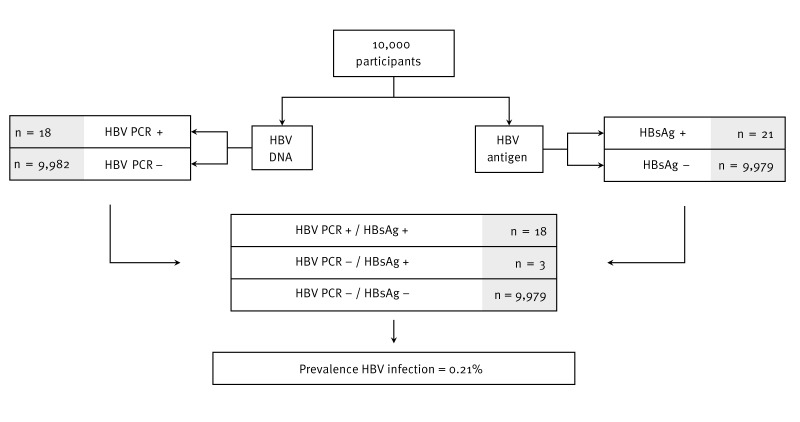
Workflow for testing of samples for hepatitis B virus from the participants of the MuSPAD cohort, Germany, 2020–2021 (n = 10,000)

HBsAg: hepatitis B surface antigen; HBV: hepatitis B virus; MuSPAD cohort: Multilocal and Serial Prevalence Study of Antibodies against SARS-CoV-2 in Germany.

Figure 3 presents the workflow for testing for HBV infection. We identified 21 participants who tested positive for hepatitis B surface antigen (HBsAg), corresponding to a prevalence of 0.21% (95% CI: 0.14–0.32) (Figure 3, Table 2 and Table 3). Eighteen of these were also positive for HBV DNA (Figure 3, Table 3). Genotyping revealed subtypes A2, C1 and D1–D4 (Table 3). Due to the low number for each genotype, we did not perform statistical analysis and the results remain descriptive. The median age of the participants infected with HBV was 53 years (range: 29–72 years). Eleven of the infected persons were women. In the cohort, the median age was 53 years (range: 18–99 years) and 55.9% were women.

**Table 3:** Demographic and virological characteristics of participants of the MuSPAD cohort infected with hepatitis B virus, Germany, 2020–2021 (n = 21)

ID	Age (years)	Gender	HBV-PCR (IU/ml)	HBsAg (S/CO)	HBV genotype	Anti HBs (mIU/mL)	Anti HBc (S/CO)	Anti HDV (AU/ml)	HDV-PCR (IU/mL)
1	40–49	Woman	3,680	4,808	D1	Negative	6.31	< 0.100	NA^a^
2	70–79	Woman	320	5,218	NA^a^	Negative	6.34	< 0.100	NA^a^
3	20–29	Man	3,720	2,158	D2	Negative	6.08	< 0.100	NA^a^
4	50–59	Woman	940	1,279	D1	Negative	6.24	< 0.100	NA^a^
5	30–39	Man	2,600	4,354	C1	Negative	6.07	< 0.100	NA^a^
6	50–59	Woman	380	3,917	A2	Negative	6.45	< 0.100	NA^a^
7	30–39	Man	3,020	5,402	A2	Negative	6.46	< 0.100	NA^a^
8	50–59	Woman	200	2,967	NA^b^	Negative	6.33	< 0.100	NA^a^
9	50–59	Man	< 10	4,974	NA^b^	Negative	5.72	< 0.100	NA^a^
10	30–39	Woman	6,840	4,649	D2	Negative	6.50	< 0.100	NA^a^
11	60–69	Woman	360	5,714	A2	Negative	5.86	< 0.100	NA^a^
12	50–59	Man	19,940	4,054	D4	Negative	5.59	< 0.100	NA^a^
13	50–59	Man	1,240	4,601	A2	Negative	6.50	< 0.100	NA^a^
14	30–39	Woman	1,180	2,488	D1	Negative	6.08	< 0.100	NA^a^
15	30–39	Man	4,900	5,843	NA^a^	NA^a^	7.36	NA^a^	NA^a^
16	30–39	Woman	256	2,691	NA^a^	NA^a^	NA	NA^a^	Negative
17	40–49	Man	452	2,529	NA^a^	NA^a^	NA	NA^a^	Negative
18	70–79	Man	372	2,117	NA^a^	NA^a^	NA	NA^a^	Negative
19	60–69	Man	Negative	1,937	NA^a^	NA^a^	NA	NA^a^	NA^a^
20	70–79	Woman	Negative	6,388	NA^a^	NA^a^	NA	NA^a^	Negative
21	30–39	Woman	Negative	6,408	NA^a^	NA^a^	NA	NA^a^	< 50^c^

HBsAg: core antigen of hepatitis B virus; HBs: surface antigen of hepatitis B virus; HBV: hepatitis B virus; HDV: hepatitis D virus; NA: not applicable; S/CO: signal/cut-off.

^a^ Limited quantity of material available.

^b^ Viral load insufficient for genotyping.

^c^ Positive within the borderline range.

Most infections with HBV were seen in 30–39-year-olds (0.44%; 95% CI: 0.22–0.91) (Figure 2B, Table 2). Of the 19 HBV-positive samples tested for HDV, one showed a borderline result that could not be confirmed because we did not have enough sample material (Table 3). No participant tested positive for both HCV and HBV.

### Overall awareness and characteristics

Retrospective testing for this study was performed in 2023/2024. Approximately 3 years after the original sample collection, hepatitis B and/or C infections in 25 persons of the study were notified to the local health departments. These people were contacted via postal mail and follow-up phone call. Of these 25 participants, four had tested positive for HCV RNA and 21 for HBV (HBsAg and/or HBV-DNA) (Table 1 and Table 2). None of the HCV-positive participants could be reached or responded to the contact attempts by the study team. Seven of the HBV-positive participants were successfully contacted and agreed to provide additional information. Three of them were unaware of their infection status, four were aware and reported currently receiving treatment (ID 2, 4, 10 and 11 in Table 3).

## Discussion

In this large population-based study, the prevalence of HCV infection was 0.04% and the prevalence of HBV infection 0.21%. At least three infections were previously undiagnosed, underlining a potential diagnostic gap despite existing screening opportunities.

Compared with the recent German Check-up 35 + programme, our study estimated a lower prevalence of undetected HBV and HCV infection [21]. In contrast, our reported prevalences were higher than those from the national blood donor surveillance [19] which likely underestimates true prevalence due to a healthier, lower-risk population. Compared with the DEGS1 survey (2008–2011) [18], which reported 0.3% prevalence for both HCV and HBV, our data suggest a decline in HCV prevalence, consistent with the impact of DAAs. For HBV, persistence in younger age groups despite vaccination points to incomplete coverage and possible migration-related exposures, which would be in line with modelling studies [31,32].

The age distribution of the persons with hepatitis infection identified in this study may point to blind spots in the current screening policies. We identified more HCV infections among older adults who are largely covered by the Check-up 35 + programme. However, a recent evaluation of the general health check-ups offered by the German health system (including the 35 + programme) found uptake to be higher among individuals who already had frequent contact with the healthcare system, but lower among populations with higher health risks and lower healthcare utilisation [22]. This suggests that while established HCV screening efforts among older adults are good, they have imperfect reach.

Meanwhile, HBV prevalence was highest among 30–39-year-olds — a group not fully reached by existing screening programmes. Expanding eligibility to adults under 35 years, particularly those with incomplete vaccination or higher exposure risks, should therefore be considered. In addition, strengthening HBV vaccination coverage, ensuring systematic HDV testing for all HBV-positive individuals and improving linkage to care for newly diagnosed patients remain essential steps towards elimination.

To achieve the WHO 2030 targets, Germany will need a two-pronged approach: sustained universal prevention (vaccination and awareness) combined with targeted testing strategies that include high-risk and under-served populations. This will be essential to close diagnostic gaps and to ensure that elimination efforts are both epidemiologically sound and socially equitable.

As many European countries similarly rely on blood donor surveillance or selected clinical cohorts, comparable gaps in detection and screening coverage are likely to exist across the region. These findings highlight the importance of population-based seroprevalence data to guide progress towards WHO elimination targets. Population-based surveillance efforts, like this one, are essential to accurately monitor progress towards hepatitis elimination targets and to ensure equitable access to diagnosis and care.

Strengths of this study include the use of a large well-characterised and geographically diverse population-based cohort, allowing more representative estimates than studies limited to blood donors or defined high-risk groups. The combined molecular and serological testing approach, validation of the pooling strategy and inclusion of HDV testing provide a comprehensive overview of viral hepatitis prevalence in Germany. While we acknowledge that retrospective testing within an existing cohort is inherently less flexible than prospectively designed studies, the use of an established well-characterised cohort—originally designed for COVID-19 research—also represents an important strength of this work. Leveraging an already funded and operational cohort enables cost-effective analyses with substantially reduced personnel and infrastructure requirements. This approach may facilitate retrospective investigations of other infectious diseases in settings with limited resources, such as non-university hospitals or institutions in lower-income countries and during periods of high system strain, including pandemics or other public health emergencies. We believe this demonstrates the potential value of repurposing existing cohorts to address additional public health questions beyond their original scope.

However, several limitations should be considered. As the MuSPAD cohort was not originally designed for HBV/HCV surveillance, detailed data on HBV vaccination status, country of birth or migration background were unavailable. Participation required proficiency in German, which likely underrepresented migrant groups, socioeconomically disadvantaged populations and individuals with limited healthcare access, groups known to have higher HBV and HCV prevalence. It should be noted that collecting detailed information on immigration status or illegal drug use may increase non-response, especially among populations that may be most relevant to the research question. Addressing these complexities would require dedicated study designs and careful methodological consideration in future research. Consequently, the unweighted estimates reported here may underestimate the true national prevalence, particularly for HBV. All prevalence estimates are presented as unweighted raw values and were not adjusted to reflect population representativeness or to diagnostic test accuracy. Due to the very small number of participants with positive test results, our estimates are unlikely to change meaningfully after weighting. Pooling and freeze–thaw cycles may have further increased the risk of false negatives, although our validation experiments minimised this risk. The low follow-up response among positive individuals also limited assessment of awareness and linkage to care. This limitation was likely exacerbated by the 3-year interval between the original sample collection and the follow-up telephone contact, which was inherent to the retrospective study design.

As a limitation we note that, like other cohorts, our population-based study may underrepresent subgroups with higher HBV/HCV prevalence, such as migrants and individuals with limited healthcare access. Integrating representative sampling of these groups into national surveillance systems, complemented with target group specific testing strategies, is critical to obtain accurate estimates and to target screening and prevention efforts to population groups with high risk and/or high burden.

Despite these constraints, this study provides post-DAA, population-based estimates for Germany and highlights key surveillance and screening gaps. Future nationwide surveillance efforts should integrate weighted analyses and targeted sampling of high-risk populations to obtain fully representative estimates for HBV and HCV elimination monitoring.

## Conclusion

Prevalence of HCV, HBV and HDV in Germany is low but not negligible. Several infected individuals were previously unaware of their status, indicating diagnostic gaps. Expanding targeted testing and ensuring systematic HDV screening in all HBV-positive individuals are essential to closing diagnostic gaps. Targeted interventions for higher-risk groups and improved linkage to care will help advance viral hepatitis elimination.

## Data Availability

All data presented in the manuscript or Supplementary Material are available from the corresponding author upon reasonable request.

## Supplementary Data

127000 Supplementary Material
