# Supplementary material for: Population-based prevalence and screening gaps of hepatitis C, B and D, Germany, 2020 to 2021
Source: Euro Surveill. 2026 Jun 25;31(25):2500864. doi: 10.2807/1560-7917.ES.2026.31.25.2500864 (PMC13309759; doi:10.2807/1560-7917.ES.2026.31.25.2500864)
Supplement: Supplementary Material [file 25-00864_CIESEK_Supplement.pdf]

Supplement information for Population-based prevalence and screening gaps of hepatitis C, B and D, Germany, 2020 to 2021

Disclaimer:

This supplementary material is hosted by Eurosurveillance as supporting information alongside the article "Population-based prevalence and screening gaps of hepatitis C, B and D, Germany, 2020 to 2021", on behalf of the authors, who remain responsible for the accuracy and appropriateness of the content. The same standards for ethics, copyright, attributions and permissions as for the article apply. Supplements are not edited by Eurosurveillance and the journal is not responsible for the maintenance of any links or email addresses provided therein.

Supplement methods

Pooling strategies, spiking and monitoring of anti-HBs IgG

Selected serum samples were shipped frozen on dry ice from the Helmholtz Centre for Infection Research, Braunschweig, Germany to the Institute of Medical Virology at the University Hospital of the Goethe-University in Frankfurt am Main, Germany, where they were stored at -80°C until further processing. The samples were organized in boxes containing 96 barcoded tubes. Samples were thawed, mixed and pooled prior to analysis. Each pool consisted of 48 individual samples (50 µl per sample, total 2400 µl; dilution 1:48), corresponding to half a plate (rows A-H, 1-6 and 7-12). Positive pools were further resolved by testing smaller sub-pools (6 samples per pool, using 50 µl per sample; dilution 1:20) and finally all individual samples from any positive sub-pool were tested separately to identify the positive sample. Positive results were confirmed by repeat testing to minimize the risk of false positives. For PCR analysis, pools were supplemented with negative human control plasma to a final volume of 1000 µl, when necessary. To ensure assay sensitivity, negative pools were initially spiked with viral genetic material to determine the appropriate dilution and confirm that detection limits were maintained. A total of 10,000 samples were distributed across 107 plates, resulting in 213 pools. For HBV and HCV PCR testing, 195 pools (91.5%) tested negative, while 21 pools (9.9%) were positive for either HBV DNA or HCV RNA. No pool was positive for

both viruses. Sixteen pools tested positive for HBV DNA, and resolution analysis identified two pools containing two HBV-positive samples each. Five pools yielded a reactive (above-LOD) signal for HCV RNA. Upon individual resolution testing, one of these was determined to be false positive. For antigen testing, further 18 pools were positive. After resolution and individual testing, we resolved 21 HBsAg and 21 anti-HCV positive samples. Due to potential vaccination of participants, there is the possibility that anti-HBs IgG is present in samples that are able to neutralize HBsAg within the pools. Thus, preliminary experiments were performed to assess the extent of this effect. In these validation experiments, we determined that the concentration of HBsAg in the pool decreases by about 50% after approximately one hour of incubation, indicating partial neutralization. To monitor this effect and ensure the validity of the results, each tested pool in the study was prepared in duplicate: in one set, a known concentration of HBsAg was spiked into the pool prior to analysis, serving as a marker for potential neutralization. By using rapid pooling procedures and prioritizing the serological analysis workflow, we assessed that the degree of HBsAg neutralization during routine testing was minimal. Consequently, the results obtained were considered reliable and negative findings were interpreted as true negatives.

HCV and HBV Genotyping

Genotyping of the positive HCV or HBV samples was performed using Sanger sequencing. Nested PCR and gel electrophoresis were performed in-house and purified PCR products were forwarded to the GLP-certified Sanger Sequencing laboratory GATC/Eurofins. Sequences were classified using the Geneious Prime software (Biomatters Ltd.) and compared with open databases for genotype classification. For HCV, the NS5B region was amplified using primers with the sequences 5′-TGG GGA TCC CGT ATG ATA CCC GCT GCT TTG A-3′, 5′-GGC GGA ATT CCT GGT CAT AGC CTC CGT GAA-3′, 5′-TAT GAY ACC CGC TGY TTT GAC TC-3′ and 5′-GCN GAR TAY CTV GTC ATA GCC TC-3′ based on the protocol described in [1]. For HBV, primer sets with the sequences 5′-ACA AGC GGC TAG GAG TTC CGC-3′, 5′-CCT CAG GCC ATG CAG TGG AA-3′, 5′-CCC AAA AGA CCC ACA ATW C-3′ and 5′-TGG TTA TCG CTG GAT GTG TC-3′ were adapted from published protocols [2,3].

Supplement Information Alinity m system for quantitative real-time PCR to test HCV-RNA or HBV-DNA

The Alinity m System was calibrated every day before routine operation, in accordance with manufacturer guidelines. The Alinity m HCV assay has a quantification range from 12 to 1 × 10^8 IU/ml (1.1–8.0 Log_{10} IU/ml) and a limit of detection (LOD) of 5.11 IU/ml for both plasma and serum [4]. The Alinity m HBV assay quantifies from 10 to 1 x10^9 IU/ml (1.0–9.0 Log_{10} IU/ml), with an LOD of 6.72 IU/ml in plasma and 9.62 IU/ml in serum, the lower limit of quantification (LLOQ) is 10 IU/ml [5]. Due to the 1:48 pooling strategy, the effective LOD increases proportionally and was estimated at 245.28 IU/ml for HCV and 461.76 IU/ml for HBV in serum. To evaluate the practical sensitivity of the pooling strategy under realistic conditions, several 48 sample pools (each consisting of 50 µl per individual sample) were experimentally spiked with low concentrations of viral nucleic acid reference material and tested using the Alinity m System. These validation experiments showed that, despite the increased detection limit due to pooling, the platform can detect low viral concentrations in pooled samples.

Supplement References:

1. Sandres-Sauné K, Deny P, Pasquier C, Thibaut V, Duverlie G, Izopet G. Determining hepatitis C genotype by analyzing the sequence of the NS5b region. Journal of Virological Methods. 2003 May;109(2):187‑93.

2. Tenney DJ, Levine SM, Rose RE, Walsh AW, Weinheimer SP, Discotto L, et al. Clinical Emergence of Entecavir-Resistant Hepatitis B Virus Requires Additional Substitutions in Virus Already Resistant to Lamivudine. Antimicrob Agents Chemother. 2004 Sep;48(9):3498‑507.

3. Thibault V, Benhamou Y, Seguret C, Bochet M, Katlama C, Bricaire F, et al. Hepatitis B Virus (HBV) Mutations Associated with Resistance to Lamivudine in Patients Coinfected with HBV and Human Immunodeficiency Virus. J Clin Microbiol. 1999 Sep;37(9):3013‑6.

4. Chevaliez S, Onelia F, Pacenti M, Goldstein E, Galán JC, Martínez-García L, et al. Multicenter clinical evaluation of alinity m HCV assay performance. Journal of Clinical Virology. 2020 Aug;129:104531.

5. Bonanzinga S, Onelia F, Jackson K, Glass A, Maree L, Krügel M, et al. Multicenter clinical evaluation of alinity m HBV assay performance. Journal of Clinical Virology. 2020 Aug;129:104514.
